# An Equivalent Structural Stress-Based Frequency-Domain Fatigue Assessment Approach for Welded Structures under Random Loading

**DOI:** 10.3390/ma16237420

**Published:** 2023-11-29

**Authors:** Uchenna Kalu, Xihui Liang

**Affiliations:** Department of Mechanical Engineering, University of Manitoba, 75A Chancellors Circle, Winnipeg, MB R3T 5V6, Canada; uchenna.j.kalu@gmail.com

**Keywords:** fatigue damage, fatigue life, welded structures, frequency domain, random loading, numerical simulation, equivalent structural stress

## Abstract

Welded structures under random loadings are usually susceptible to fatigue-induced failures that lead to significant economic and safety effects. However, accurately predicting these structures’ fatigue damage and life in the frequency domain remains challenging due to the limitations associated with using traditional weld stress extrapolation methods, such as nominal, hotspot, and notch stress methods. These methods struggle with precisely defining and characterizing the stresses at the weld toe and root as they vary depending on factors like weld stress concentration effects, joint geometry, and loading modes. This research introduces an Equilibrium Equivalent Structural Stress (EESS)-based frequency-domain fatigue analysis approach for welded structures subjected to random loading. The proposed method utilizes the EESS formulations, which are based on the decomposition and characterization of weld toe stresses with a single stress parameter, together with incorporating structural dynamic properties’ effects on the stresses acting on the weld joints and the corresponding accumulated fatigue damage of the structure. The numerical demonstration and validation of the proposed method have been performed using a welded Rectangular Hollow Section (RHS) T-joint structure subjected to stationary random fatigue loading. The proposed method’s fatigue damage and life results are compared with the fatigue test data and the equivalent hotspot stress extrapolation-based technique results.

## 1. Introduction

Welding is the most popular and preferable technique for connecting parts of steel structures in various industries. Welded connections are preferred over bolted and riveted connections in steel structures due to their unique characteristics of low cost, aesthetics, high strength-to-weight ratio, geometrical flexibility, and, most importantly, high resistance to fatigue loadings [1]. However, the complex nature of the welding process—usually associated with induced defects (e.g., slag inclusion, porosity, lack of fusion), residual stresses, microstructural distortion around the heat-affected zone, and notch-based stress concentrations—implies that the fatigue resistance at the welded connections regions are always inferior in comparison to the other unwelded areas of the structure [2]. Hence, the increased propensity of a structure to fatigue damage failure at stress levels well below its material yield strength—leading to severe economic and, in some cases, fatal consequences.

Stress-based, strain-based, or linear fracture mechanics analysis approaches can be used in Finite Element Analysis (FEA) to evaluate the fatigue life of welded structures at the different damage failure stages [3]. The stress-based method, which is also known as high-cycle fatigue and is characterized by stresses within the elastic region of the material, requires the cyclic load exposure history category, stress at the welded joint locations, and a linear or nonlinear damage prediction model that compares the evaluated cyclic stresses *S* to the material’s number of cycles to failure properties (life) *N* [4]. The stress–life (*S–N*) cycle curves of welded structures are obtained from experimental data based on a series of constant amplitude cyclic load tests for different welded joint geometry categories and loading modes.

The fatigue loadings of welded structures, categorized according to their distinctive characteristics, mainly consist of constant amplitude cyclic, variable amplitude cyclic, and random loadings [5,6,7]. The time-domain or frequency-domain fatigue analysis approaches can be used to evaluate the fatigue damage and life of welded structures exposed to random loadings [6]. In the frequency-domain approach, a structure’s loads and stress responses are considered in the Power Spectral Density (PSD) form [7]. The total fatigue damage of a structure is calculated by accumulating the damage caused by each stress spectral component using any stochastic fatigue models.

As already stated, to determine the fatigue life of welded structures using the frequency-domain fatigue analysis approach, the complete stress state within and around the welded joints is required; thus, they must be evaluated. Traditional methods for evaluating the weld toe stresses include nominal weld stress, hotspot weld stress, and notch weld stress evaluation methods [8,9,10]. Studies and research on fatigue analysis of welded structures in the frequency domain [6,11,12] use traditional stress evaluation methods in evaluating the weld toe stress PSD.

The weld toe stresses evaluated using the nominal stress extrapolation are extracted from the cross-sections of the structure away from the weld toe region. These stresses do not include the local stress concentrations due to the weld toe notches and joint geometry raiser effects—already included in the *S–N* curves [10]. Thus, this method is only suitable for structures with simple joint geometry with no combined loading modes [8]. The hotspot stress extrapolation method is an improvement on the nominal stress extrapolation method and is the most widely used traditional weld stress evaluation method. It provides a more accurate stress value by including stress raiser effects due to welded joint geometry and notches that are not categorically defined by relevant standards and disregarded in the nominal stress extrapolation method [10]. In [11], this stress evaluation method was utilized to evaluate the multiaxial random stresses, residual stress, and mean stress effects at the weld joint of various welded components and structural systems. However, it excludes the nonlinear peak stress caused by the local weld toe notches and stress distribution across the thickness of the structural components. This method is suitable for simple and complex structures. Still, the dependence of the calculated stress concentrations on the unique welded joint geometry detail, loading modes, and quality of the finite element model mesh characteristics limit its utilization. The notch stress extrapolation method, an improvement over the nominal and hotspot stress extrapolation methods, includes all stress raiser effects due to geometrical discontinuities, weld toe, and root notches. However, it excludes the peak stress effect due to the welded joint geometry already accounted for in the experimental S–N curve test data [9,13]. Local sub-modelling and extreme mesh refinement are required to use this method in finite element fatigue analysis. Hence, applying this method in large and complex structures becomes practically impossible.

An alternative weld stress evaluation method, called the Equilibrium Equivalent Structural Stress (EESS) [14,15,16,17], characterizes the decomposed weld toe stress concentrations that are influenced by the weld thicknesses, loading modes, and joint geometries, using an equivalent stress-based parameter. Thus, it enables mesh-insensitive finite element analysis and improves the accuracy of stress and fatigue life results. It also consolidates a large amount of S–N test data into a single narrow scatter band master S–N curve [18,19]. The master S–N curve is derived from combining many experimental S–N curve data for various categories of welded structures, thereby overcoming the challenges and limitations associated with the use of traditional weld stress evaluation methods. This method has primarily been used to analyze welded structures’ fatigue behavior and life under constant amplitude cyclic uniaxial [20,21,22,23] and multiaxial [24,25] loads. Researchers have demonstrated the efficacy and robustness of the method for single-spot and multiple-spot welded structures [26,27,28]. Seam-welded structures exposed to variable amplitude loading conditions [29,30] have also been evaluated using the time-domain fatigue analysis approach based on the path-dependent maximum range approach. However, there is currently no established method for applying the EESS method to evaluate fatigue responses of randomly loaded welded structures whose fatigue behavior and life are predicted using the frequency-domain fatigue analysis approach.

This research proposes a new frequency-domain fatigue analysis method for evaluating and predicting the fatigue damage and life of welded structures that are subjected to random fatigue loads. The method, aimed at evaluating the damage and life at crack initiation, incorporates an EESS response PSD that is mesh insensitive and characterized by a stress concentration parameter with the master S–N curve. The proposed EESS equations and EESS-based frequency-domain fatigue analysis procedure are numerically implemented and validated using a welded Rectangular Hollow Section (RHS) structure that is subjected to road surface profile-induced ergodic stationary random loadings. In addition, the implications and significance of the fatigue stress, damage, and life results are discussed in detail and used to validate the proposed methodology results with experimental fatigue damage data in the literature and hotspot Equivalent Von-Mises (EQVM) stress method results for further interpretations and potential variability to existing fatigue evaluation methods. The proposed method encompasses a multifaceted approach that considers the equations, theories, and principles of the EESS constant cyclic amplitude-loading fatigue analysis method for welded structures, frequency-domain fatigue analysis equations and models for randomly loaded structures, and dynamic response characteristics and equations for dynamically excited structures.

## 2. Frequency-Domain Fatigue Damage and Life Evaluation Method

The frequency-domain fatigue analysis considers a structure’s stationary random loadings and the resulting stress response in their condensed PSD form. A structure’s dynamic behaviour and response to random loadings can be mainly governed by its Frequency Response Function (FRF). The fatigue damage is subsequently assessed by accumulating the damage caused by each spectral stress component across the loading frequency range using stochastic fatigue models.

Analytical equations and procedures are defined and developed to evaluate the stress response PSD at the weld joints, considering the specific characteristics of the weld toe stress concentrations, structural component thickness, and loading modes within the welded structure. The quasi-static equations and theories of the EESS methodology and some fundamental structural dynamics principles that form the basis of the proposed method are briefly introduced in Section 2.1 and Section 2.2.1.

### 2.1. Equilibrium Equivalent Structural Stresses at Welded Joints

**Figure 1:**
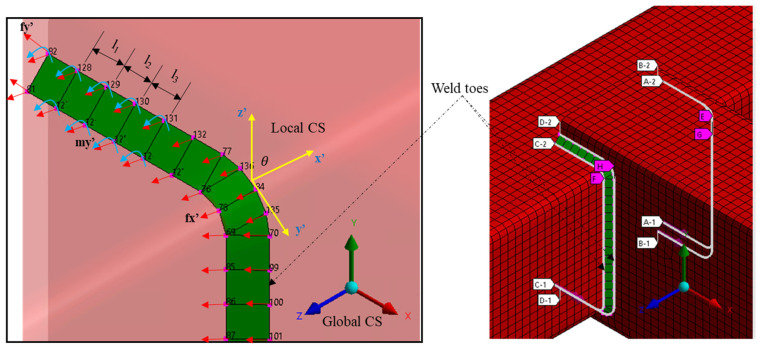
Structural stress definition and calculation from finite element model mesh elements.

The EESS fatigue assessment method for welded structures, developed by Dong in 2001 [16], involves computing structural stresses from the decomposition of the peak stresses at the weld toe into balanced primary membrane stress $\sigma_{m}$ bending stress $\sigma_{b}$ and self-equilibrating nonlinear stress $\sigma_{nl}$, as shown in Figure 1.

The weld plane’s normal structural stress $\sigma_{s}$, parallel shear structural stress $\tau_{z}$, and perpendicular shear structural stress $\tau_{s}$ are calculated from the mesh nodes as shown in Figure 1 using Equations (1)–(3) [16,18]. The stresses are based on the decomposed primary membrane and bending stresses.

(1) $$\sigma_{s}=\sigma_{m}+\sigma_{b}=\frac{f_{x}^{\prime }}{t}+\frac{{6m}_{y}^{\prime }}{t^{2}}$$

(2) $$\tau_{s}=\tau_{m}+\tau_{b}=\frac{f_{y}^{\prime }}{t}+\frac{{6m}_{x}^{\prime }}{t^{2}}$$

(3) $$\tau_{z}=\frac{f_{z}^{\prime }}{t}$$

where t represents the weld thickness, and $\sigma_{m}$ and $\sigma_{b}$ are the normal membrane and bending stresses extrapolated from the welded joints, respectively. The corresponding shear membrane and bending stresses are given by $\tau_{m}$ and $\tau_{b}$, respectively. $f_{x}^{\prime },f_{y}^{\prime }$, and $f_{z}^{\prime }$ are the line forces along the weld mesh element’s local x-axis, y-axis, and z-axis coordinate system, respectively, considering each adjacent node’s contribution. $m_{x}^{\prime },m_{y}^{\prime }$ and $m_{z}^{\prime }$ are the corresponding line moment values.

### 2.2. Establishment of the Weld’s Dynamic Equivalent Stress and Fatigue Damage

#### 2.2.1. Structural Systems Dynamics

Welded structures exhibit dynamic behavior when subjected to random loadings that have frequency spectrums close to the natural frequencies of the structures. This phenomenon necessitates analyzing and describing these structures as dynamic systems. To adequately capture the dynamic response of Multiple Degree-Of-Freedom (MDOF) linear structures, with the consideration of their dynamic characteristics (stiffness, damping, and inertia), the equation of motion in the physical coordinates, as represented by Equation (4) [31,32], is employed.

(4) $$M\ddot{u}(t)+jC\dot{u}(t)+Ku(t)=f(t)$$

where ***f****(t)* represents the applied excitation load vector acting on the structure, *M* denotes the inertia matrix, C represents the viscous damping matrix, and *K* is the structural stiffness matrix. $\ddot{u}$(*t*), $\dot{u}($*t*), and ***u***(*t*) are the acceleration, velocity, and displacement vector variables, respectively.

The coupled equations of motion for the MDOF system given in Equation (4) can be expressed in the modal coordinates by Equations (5)–(7) [33,34], which is based on the Mode Superposition (MSUP) theory and considering an initial displacement mode shape.

(5) $${-\omega^{2}\emptyset }^{T}M\emptyset \eta (\omega )+{j\omega \emptyset }^{T}C\emptyset \eta (\omega )+\emptyset^{T}K\emptyset \eta (\omega )=\emptyset^{T}F(\omega )$$

(6) $$\emptyset =\left[\varphi_{1},{\;\varphi }_{2},\;\varphi_{3},\ldots \varphi_{n}\right]$$

(7) $$\eta =\left[\eta_{1},\eta_{2},\;\eta_{3},\ldots \eta_{n}\right]$$

where $\emptyset^{T}$ is the transpose of the mode shapes matrix. ${M=\emptyset }^{T}M\emptyset$ represents the modal mass matrix, ${C=\emptyset }^{T}C\emptyset$ denotes the modal viscous damping matrix, ${K=\emptyset }^{T}K\emptyset$ is the modal stiffness matrix, and $\emptyset^{T}F(\omega )$ is the modal force vector.

The uncoupled form of Equation (5), consisting of a set of modal Single Degree of Freedom (SDOF) equations in the modal coordinate system, is given by Equation (8) [34].

(8) $$-{\omega^{2}m}_{i}\eta_{i}(\omega )+{j\omega c}_{i}\eta_{i}(\omega )+k_{i}\eta_{i}(\omega )=f_{i}(\omega )\;$$

where $m_{i}$, $c_{i}$, $k_{i}$, and $f_{i}$ are the $i^{th}$ modal mass, $i^{th}$ modal viscous modal damping, $i^{th}$ modal structural stiffness, and $i^{th}$ modal force vector respectively corresponding to a structure’s $i^{th}$ modal response.

From Equation (8), the displacement vector of a randomly excited linear structure can be computed in the physical coordinate system by relating the individual displacement mode shapes to the modal coordinate using Equation (9) [32,33]. Equation (9) considers each mode’s contribution to the overall displacement of the structure.

(9) $$U\left(x,t\right)=\sum_{i=1}^{n}\emptyset_{i}\left(x\right)\eta_{i}\left(t\right)$$

where U(x,t) is the displacement response of the structure at location x and time t.$\;{\emptyset (x)}_{i}$ represents the $i^{th}$ displacement mode shape at location x along the structure, $\eta_{i}$ denotes the time-based modal coordinate defining the contribution of each mode shape i to the overall displacement, and n is the maximum number of modes considered in the analysis.

#### 2.2.2. Modal Equivalent Structural Stress

Following the establishment of the structural dynamic’s principles in Section 2.2.1 and the quasi-static EESS equations in Section 2.1, a modal EESS analytical equation for evaluating the modal stresses of welded structures can be developed. The linear structural response behavior considerations in [32,33], the static EESS equations given by Equations (1)–(3), and the MSUP displacement response expression, Equation (9), are considered as the basis for developing the modal EESS equations.

Consider Equations (10)–(12) [16], which are also used in [35] to extract the balanced force and moment vectors from the mesh elements nodes of the discretized finite element model of a welded structure in a linear static FEA analysis.

(10) $$\left[F_{e}\right]=\left[F_{e(x)},\;{\;F}_{e(y)}{,\;F}_{\left(z\right)},\;M_{\left(x\right)},\;M_{\left(y\right)},\;M_{\left(z\right)}\right]$$

(11) $$\left[K\right]=\left[N\times N\right]$$

(12) $$\left[U\right]=\left[u_{x},\;u_{y}{,\;u}_{z},\;\theta_{x},\;\theta_{y},\;\theta_{z}\right]$$

where $\left[F\right]$ denotes the balanced internal global force and moment vectors acting on all the nodes along the weld paths of a welded joint in the fixed global coordinate system, $\left[K\right]$ is the structure’s stiffness related to the mesh element global stiffness matrix with dimensions N×N, and $\left[U\right]$ represents the finite element weld’s node translation $u_{x},\;u_{y}{,\;u}_{z}$ and rotational $\theta_{x},\;\theta_{y},\;\theta_{z}$ displacement vector matrix with dimensions N×1. The displacement vector is dependent on the number of DOFs of the nodes. N is the number of degrees of freedom of the structure.

From Equations (9)–(12), for a dynamic welded structure, the modal response of the balanced global forces and moments acting in the global coordinate system of the weld’s discretized finite element model can be calculated based on the superposition of all the modal forces and moments corresponding to the various modal responses of the structure using Equation (13) and (14).

(13) $$\left[F_{e}\right]=\left[K\right]\left[U\right]=\left[K\right]\sum_{i=1}^{n}\emptyset_{i}\left(x\right)\eta_{i}(t)=\left(\left[K\right]\sum_{i=1}^{n}\emptyset_{i}\left(x\right)\right)\left(\sum_{i=1}^{n}\eta_{i}(t)\right)\;$$

(14) $${\emptyset_{i}}^{F_{e}}=\left[K\right]\emptyset_{i}\left(x\right)$$

where $\left[F_{e}\right]$ is considered as the total modal global force and moment vector acting on any given finite element mesh node along the weld toe paths of the structure. ${\emptyset_{i}}^{F_{e}}$(${\emptyset_{i}}^{f_{e(x)}},\;{\emptyset_{i}}^{f_{e(y)}},{\emptyset_{i}}^{f_{e(z)}},{\emptyset_{i}}^{m_{e(x)}},\;{\emptyset_{i}}^{m_{e(y)}},\;{\emptyset_{i}}^{m_{e(z)}}$) represents the modal global force and moment vector corresponding to the structure’s $i^{th}$ modal response, n denotes the number of modes considered in evaluating the modal forces and moments, and $\eta_{i}$ is the $i^{th}$ modal coordinate.

The corresponding modal mesh element line forces and moments along the weld paths of the structure can be determined from Equations (15) and (16). Both equations are derived from the transformation Equation (13) by considering the balanced global forces and moments vector, element line forces, and moment vector and transformation matrix given by the expressions of Equations (17)–(19), respectively.

(15) $$\left[f^{\prime }\right]=\left[T\right]\left[L\right]\left[K\right]\sum_{i=1}^{n}\emptyset_{i}\eta_{i}$$

(16) $${\emptyset_{i}}^{f^{\prime }}=\left[T\right]\left[L\right]\left[K\right]\emptyset_{i}$$

where ${\emptyset_{i}}^{f^{\prime }}$(${\emptyset_{i}}^{f_{x}^{\prime }},{\emptyset_{i}}^{f_{y}^{\prime }},{\emptyset_{i}}^{f_{z}^{\prime }},{\emptyset_{i}}^{m_{x}^{\prime }},{\emptyset_{i}}^{m_{y}^{\prime }},{\emptyset_{i}}^{m_{z}^{\prime }}$) represents the modal line force and moment vector corresponding to the $i^{th}$ modal response of the structure.

(17) $$\left[f^{\prime }\right]=\left[F\right]\left[L\right]$$

(18) $$\left[F\right]=\left[F_{e}\right]\left[T\right]$$

(19) $$\;\;\;\;\;\left[T\right]=\left[\begin{array}{ccc} cos\theta_{x^{\prime }X} & sin\theta_{y^{\prime }Y} & 0\\ sin\theta_{x^{\prime }X} & cos\theta_{y^{\prime }Y} & 0\\ 0 & 0\; & cos\theta_{z^{\prime }Z} \end{array}\right]$$

where $\left[f^{\prime }\right]$ denotes the local line forces and moments acting on the weld’s mesh elements, $\left[F\right]$ is the balanced internal force and moment vectors acting on all the nodes along the weld paths in the element’s local coordinate, and $\left[L\right]$ represents the matrix of mesh element lengths representing a modelled weld detail based on considering a continuous weld toe line along the mesh nodes [22,24]. The continuous weld toe line ensures that no abrupt stress intensities increase at the weld ends, leading to a violation of the EESS principles for weld’s stress insensitivity to finite element model mesh properties. $\left[T\right]$ is the transformation matrix of sines and cosines angles between the structure’s global coordinate system and the mesh elements’ local coordinate system.

Once the equations for estimating the modal line forces and moments are established, the dynamic modal normal and shear structural stresses for all the mesh elements’ nodes along the weld path detail can be calculated using Equations (20)–(22). These equations, which consider all the mode shapes appropriately defining the structure’s response, are derived from the static structural stress equations, Equations (1)–(3), with the element’s line modal force and moment equation, Equation (15).

(20) $${\emptyset_{i}}^{\sigma_{∆s}}=\frac{{\emptyset_{i}}^{f_{x}^{\prime }}}{t}+\frac{6{\emptyset_{i}}^{m_{y}^{\prime }}}{t^{2}}={\emptyset_{i}}^{\sigma_{m}}+{\emptyset_{i}}^{\sigma_{b}}\;$$

(21) $${\emptyset_{i}}^{\tau_{∆s}}=\frac{{\emptyset_{i}}^{f_{y}^{\prime }}}{t}+\frac{6{\emptyset_{i}}^{m_{x}^{\prime }}}{t^{2}}={\emptyset_{i}}^{\tau_{m}}+{\emptyset_{i}}^{\tau_{b}}$$

(22) $${\emptyset_{i}}^{\tau_{∆z}}=\frac{{\emptyset_{i}}^{f_{z}^{\prime }}}{t}$$

where ${\emptyset_{i}}^{\sigma_{∆s}},\;{\emptyset_{i}}^{\tau_{∆s}}$ and ${\emptyset_{i}}^{\tau_{∆s}}$ are the modal normal, modal perpendicular shear and modal parallel stresses relating to the structure’s $i^{th}$ modal response. ${\emptyset_{i}}^{\sigma_{m}}$ and ${\emptyset_{i}}^{\sigma_{b}}$ are the modal normal membrane and bending stresses, while ${\emptyset_{i}}^{\tau_{m}}$ and ${\emptyset_{i}}^{\tau_{b}}$ are the corresponding modal shear components.

The modal EESS, which offers a more accurate representation of the stress distribution, considering the specific characteristics of the weld toe stress concentrations, structural component thickness, and loading modes within the welded structure, can be calculated using Equations (23)–(25), which is derived from Equation (20) and the loading mode correction factor ${I(r)}^{1/m}$ in [19].

(23) $${\emptyset_{i}}^{{∆S}_{s}}=\frac{{\emptyset_{i}}^{\sigma_{∆s}}}{t^{*\frac{2-m}{2m}\;}\cdot {\emptyset_{i}}^{{I(r)}^{1/m}}}$$

(24) $${\emptyset_{i}}^{{I(r)}^{1/m}}={0.0011r}^{6}+{0.0767r}^{5}-{0.0988r}^{4}+{0.0946r}^{3}+{0.0221r}^{2}+0.014r+1.223\;$$

(25) $$r=\frac{\left|{\emptyset_{i}}^{\sigma_{b}}\right|}{\left|{\emptyset_{i}}^{\sigma_{m}}\right|+\left|{\emptyset_{i}}^{\sigma_{b}}\right|}$$

where ${\emptyset_{i}}^{{I(r)}^{1/m}}$ represents the load correction factor for the $i^{th}$ mode, and m and $t^{*}$ taken as 1 mm are the hypothetical crack depth and reference plate thickness, respectively.

#### 2.2.3. PSD of Equivalent Structural Stress

In the frequency domain, a structure’s steady-state stress Frequency Response Function (FRF) can be computed using Equation (26). This equation, which is based on the modal coordinate FRF, considers the relationship between the dynamic displacement response, U(x,t), the strain response, ε(x,t) and the stress response, σ(x,t) at a specific location x within the structure and at a given time t [31,34].

(26) $$H^{\sigma }\left(\omega \right)=\sum_{i=1}^{n}{\emptyset_{i}\left(x\right)}^{\sigma }\eta_{i}\left(t\right)=\;\sum_{i=1}^{n}\frac{{\emptyset_{i}\left(x\right)}^{\sigma }\emptyset_{i}^{T}}{{{[-\omega }^{2}m}_{i}+{j\omega c}_{i}+k_{i}]}\;\;\;\;\;\;\;\;\;\;\;\;\;\;\;\;\;$$

(27) $${\emptyset_{i}\left(x\right)}^{\sigma }=E.{\emptyset_{I}\left(x\right)}^{\varepsilon }$$

(28) $${\emptyset_{i}\left(x\right)}^{\varepsilon }=D.\emptyset_{i}\left(x\right)$$

where $H^{\sigma }\left(\omega \right)$ denotes the total mode-based stress FRF at a location x and time t on the structure. It relates the input random PSD loading to the output stress PSD. ${\emptyset_{i}\left(x\right)}^{\sigma }$ represents the $i^{th}$ stress mode shape at location x, which is typically a hotspot normal or shear stress mode shape for welded structures, with *E* being the modulus of elasticity. ${\emptyset_{i}\left(x\right)}^{\varepsilon }$ denotes the $i^{th}$ strain mode shape at location x, with *D* being the differential operator. $\eta_{i}$ is $i^{th}$ modal coordinate FRF.

The model-based stress FRF of a harmonically excited dynamic welded structure, accounting for the influence of the EESS in the weld joint area, can be obtained by summing the product of the individual modal coordinates FRF $\eta_{i}\left(\omega \right)$ and the corresponding individual modal EESS parameter ${\emptyset_{i}}^{{∆S}_{s}}$ given by Equation (23) within the selected frequency range of interest.

(29) $$H^{{∆S}_{s}}\left(\omega \right)=\sum_{i=1}^{n}\frac{{\emptyset_{i}}^{{∆S}_{s}}\emptyset_{i}^{T}}{{{[-\omega }^{2}m}_{i}+{j\omega c}_{i}+k_{i}]}\;\;\;\;\;\;\;\;\;\;\;\;\;\;\;\;\;$$

where $H^{{∆S}_{s}}\left(\omega \right)$ is the EESS-based FRF, and ${\emptyset_{i}}^{{∆S}_{s}}$ denotes the modal EESS parameter.

With the mode-based EESS FRF evaluation equation established, the EESS stress PSD response of a randomly loaded welded structure can be computed based on the relationship between the input load PSD and the FRF using Equation (30).

(30) $$\left[G^{{∆S}_{s}}\left(f\right)\right]={\left[H^{{∆S}_{s}}\left(f\right)\right]}^{*}\cdot \left[G\left(f\right)\right]\cdot {\left[H^{{∆S}_{s}}\left(f\right)\right]}^{T}$$

where $G^{{∆S}_{s}}\left(f\right)$ represents the EESS PSD response, $G\left(f\right)$ is the random input load PSD, and ${H^{{∆S}_{s}}\left(f\right)}^{*}$, and ${H^{{∆S}_{s}}\left(f\right)}^{T}$ are the complex conjugate and transpose of the structure’s EESS FRF.

#### 2.2.4. Fatigue Damage and Life Estimation

Once the stress response PSD is determined, the fatigue damage and corresponding life of welded structures at the crack initiation stage can be estimated in the frequency domain using Equation (31). Equation (31) is derived by applying Dirlik’s method [33] and considering the PSD of the EESS $G^{{∆S}_{s}}\left(f\right)$ from Equation (30), mode-based EESS amplitudes Probability Density Function (PDF), and the allowable number of cycles to failure N parameter obtained from the master S–N.

(31) $$D=\frac{{E(P)}^{{∆S}_{s}}T}{C^{1/h}}\int_{0}^{+\infty }\;{p(∆S}_{s})\cdot {\left(S^{{∆S}_{s}}\right)}^{1/h}\;ds$$

(32) $$\;\;\;\;\;\;\;{p(∆S}_{s})=\frac{\frac{D_{1}}{Q}\cdot e^{\frac{-Z}{Q}}+\frac{D_{2}\cdot Z}{R^{2}}\cdot e^{\frac{-Z^{2}}{2.R^{2}}}+D_{3}\cdot {Z\cdot e}^{\frac{-Z^{2}}{2}}}{2\cdot \sqrt{m_{0}}}$$

where ${p(∆S}_{s})$ is the EESS PDF, and C and h are the master S–N curve parameters. $S^{{∆S}_{s}}$ represents the EESS stress amplitude, ${E(P)}^{{∆S}_{s}}$ is the expected number of peaks crossing per second from the EESS response PSD output, T denotes the total load exposure time for the damage, and ds is the incremental stress. $D_{1},D_{2},\;D_{3},\;R,\;Q,\;Z$ and $m_{0}$, the parameters in Dirlik’s formulation are modified based on the EESS spectral moments.

**Figure 2:**
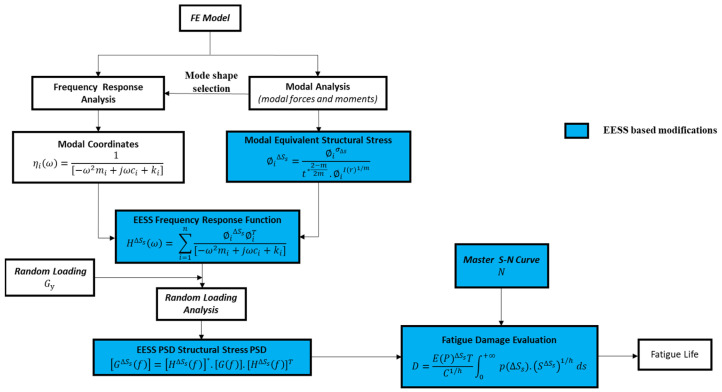
Flowchart of the frequency-domain fatigue analysis method based on the EESS.

The flowchart in Figure 2 provides a clear overview of the computational methodology employed in this research. It emphasizes the integration of the proposed EESS-based considerations in the frequency-domain fatigue analysis approach for randomly loaded welded structures. The main inputs and steps depicted by the blue boxes involve:

1. Evaluation of the modal EESS ${\emptyset_{i}}^{{∆S}_{s}}$ along the weld joints paths of the structure using Equation (23), considering the modal line element forces ${\emptyset_{i}}^{f_{x}^{\prime }}$ and moments ${\emptyset_{i}}^{m_{y}^{\prime }}$
2. Evaluation of the EESS (FRF) $H^{{∆S}_{s}}\left(\omega \right)$ at the exact weld locations as the modal EESS using Equation (29) considering the modal coordinates frequency response analysis of the structure and the calculated modal EESS ${\emptyset_{i}}^{{∆S}_{s}}$ from step (i)
3. Calculating the EESS Power Spectral Density (PSD) $G^{{∆S}_{s}}\left(f\right)$ based on the solution from step (2) and the input load PSD $G\left(f\right)$
4. Estimating the fatigue damage D due to EESS PSD at the weld joints using Equation (31) and also considering the master S–N curve parameters

## 3. Numerical Demonstration and Validation

The viability of the proposed frequency-domain fatigue analysis method to estimate and predict the fatigue damage and life of welded structures is numerically demonstrated and validated using a Rectangular Hollow Section (RHS) T-joint welded structure that is subjected to a random loading.

### 3.1. Finite Element Model

**Figure 3:**
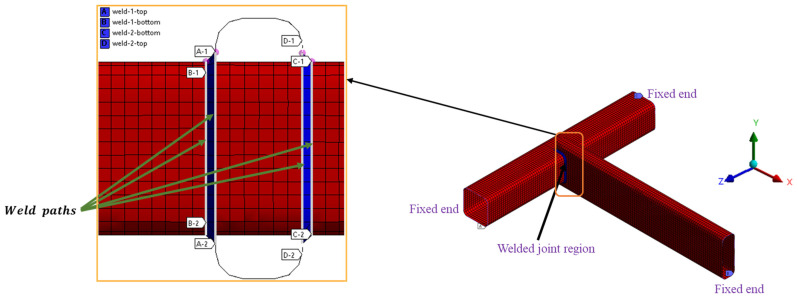
Finite element model and boundary condition of the structure.

To accurately analyze the weld joints and transfer the loads to the desired locations, this research adequately represents the structural components and weld details using Shell 181 elements, as shown in Figure 3. The structure’s mesh characteristics consist of 8262 elements and 8558 nodes. The boundary conditions include complete constraints on translation and rotation in all degrees of freedom at the ends of the structural beam components to accurately simulate the actual physical condition of the structure.

The weld details are represented by a row of inclined plate shell elements at a *45*-degree angle to accurately capture the welds’ geometry, stiffness, and strength properties at the joint connection locations following the recommendation by Hobbacher in [36]. The weld paths, which represent the actual weld toe and surface regions, are defined in the model. Four weld paths are considered in the analysis, where stresses and other fatigue variables are computed for the structure.

#### 3.1.1. Calculating the Dynamic Stress Response

The modal stresses ${\emptyset_{i}}^{{∆S}_{s}}$ at the weld joints are calculated for each mode of the structure using a developed MATLAB algorithm that is based on Equation (23) and the procedures outlined in Section 2.2. The required global modal node forces ${\emptyset_{i}}^{F_{e}}$ and moments ${\emptyset_{i}}^{M_{e}}$ are extracted from the structure’s finite element frequency response analysis solution for all considered modal responses. The Lanczos mode extraction method, which does not miss roots, supports sparse matrix, and is computationally efficient, is used to obtain all the mode shapes and natural frequencies [37].

The solutions of the modal EESS ${\emptyset_{i}}^{{∆S}_{s}}$ is combined with the modal coordinates $\eta_{i}\left(\omega \right)$ using the Equation (29) to calculate the EESS FRF of the structure for all modal responses and frequency ranges due to the unit loading excitation. This calculation is performed in a MATLAB program to evaluate the EESS FRF $H^{{∆S}_{s}}\left(\omega \right)$ of the structure. To obtain the modal coordinates responses $\eta_{i}\left(\omega \right)$, a frequency response analysis is conducted in ANSYS using the pre-stressed modal analysis finite element model. The model is subjected to a unit magnitude harmonic acceleration loading of $1\;m^{2}/s$ with a frequency spectrum ranging from 0–2000 Hz in the vertical direction (Y-axis). This frequency range covers all the considered modes and natural frequencies of the structure and is in the same direction and quantity as the excitation loading. A constant damping ratio ζ of 2% is considered to simulate the structure’s response.

**Figure 4:**
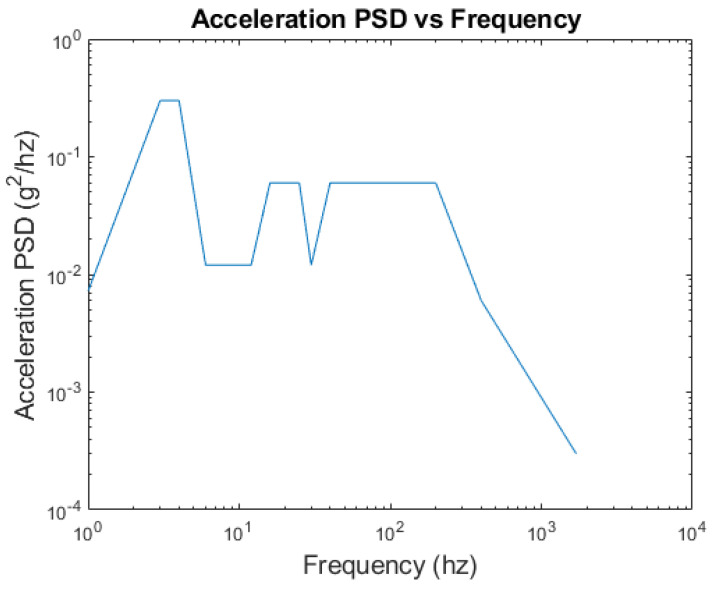
Input base acceleration PSD load in the time-domain and frequency-domain form.

**Figure 5:**
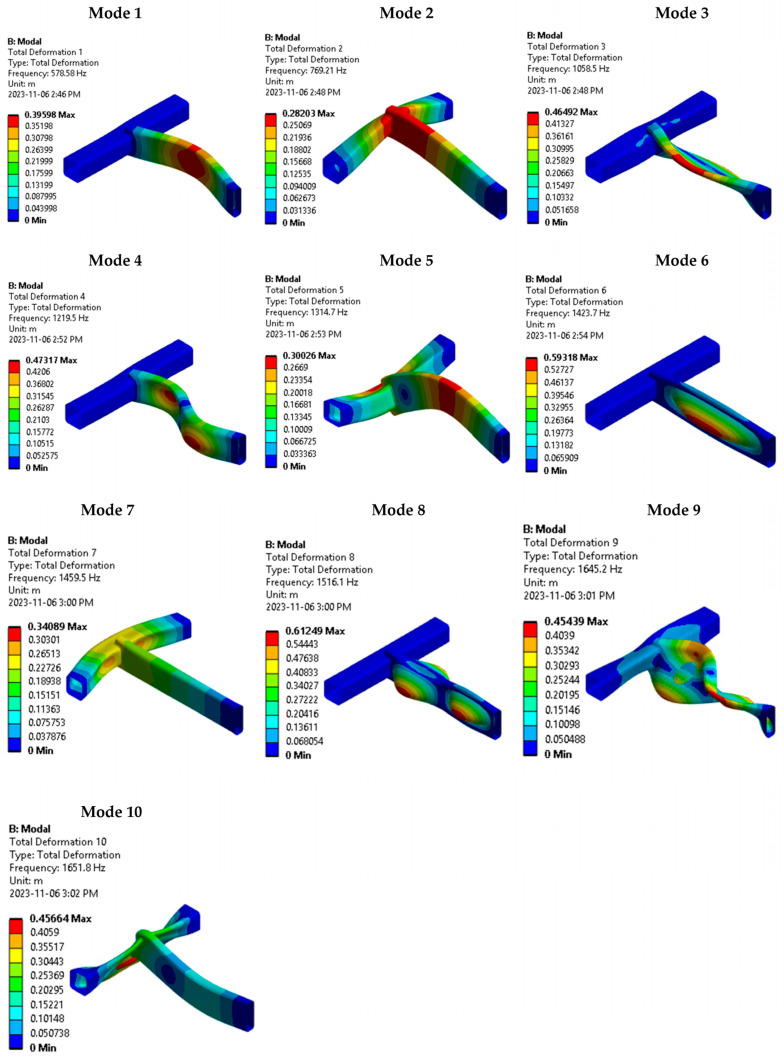
Mode shapes contour and corresponding natural frequencies of the first ten modes of the structure.

Once the EESS FRF $H^{{∆S}_{s}}\left(\omega \right)$ is calculated, it is combined with the input loading PSD loading $G\left(f\right)$ using Equation (30) to compute the EESS PSD $G^{{∆S}_{s}}\left(f\right)$ at the weld joints. This calculation is part of a frequency-domain fatigue analysis procedure executed in the MATLAB program. To determine the EESS PSD, the structure is subjected to a 1-h exposure duration of uniaxial stationary random base acceleration, representing an irregular road surface profile [38]. This loading is shown in Figure 4 and Figure 5 in the PSD frequency-domain form. The PSD loading is applied to the structure, and the resulting response is used to compute the EESS PSD response at the weld joints.

The load, with a zero mean and a wide range of frequencies from 1–1700 Hz, covering and exciting all the dominant main modes and natural frequencies of the structure, is applied in the vertical direction of the structure. The dynamic stress response PSD is computed for all the nodes along the weld paths of the structure. By importing the calculated FRF and the specified PSD loading into ANSYS ncode design life glyph works software (2022R2), the structure’s stress response distribution within the welds is analyzed and visualized.

Having computed the EESS PSD $G^{{∆S}_{s}}\left(f\right)$, the EESS amplitudes $S^{{∆S}_{s}}$ and related PDF ${p(∆S}_{s})$ parameters are evaluated. Once these parameters are calculated, the fatigue damage and life of the structure at crack initiation are subsequently assessed using Equation (31) and the procedural considerations outlined in Section 2.2.2.

#### 3.1.2. Estimating the Fatigue Damage and Life

Having computed the EESS PSD $G^{{∆S}_{s}}\left(f\right)$, the EESS amplitudes $S^{{∆S}_{s}}$ and related PDF ${p(∆S}_{s})$ parameters, the fatigue damage and life of the structure are subsequently evaluated using Equation (31). The master S–N fatigue curve slope C and intercept h parameters properties in [18] and the procedures outlined in Section 2.2.2 are utilized for the computation.

### 3.2. Results and Discussions

#### 3.2.1. Modal Response of the Structure

The displacement mode shapes modal analysis results for the first ten selected modes with their associated natural frequencies are presented in Figure 5. These mode shapes provide insights into the structural response at different vibration frequencies.

By analyzing the participation factors of the contributing modes, it is determined that modes 2, 5, and 10 exhibit in-plane displacement mode shapes. These mode shapes are aligned with the applied base acceleration excitation and are the dominant modes. These modes significantly influence the structure’s response and contribute substantially to the accumulated fatigue damage. The subsequent stress response and damage life evaluations consider all the first ten modes.

**Figure 6:**
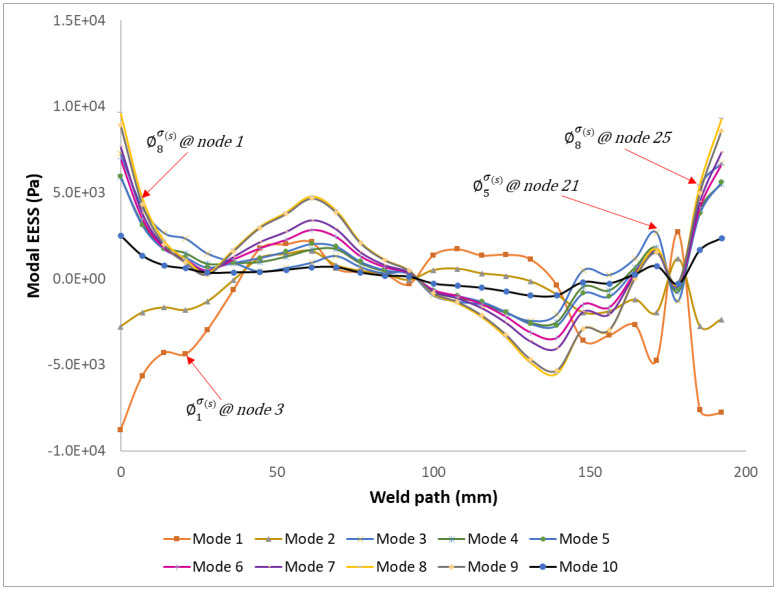

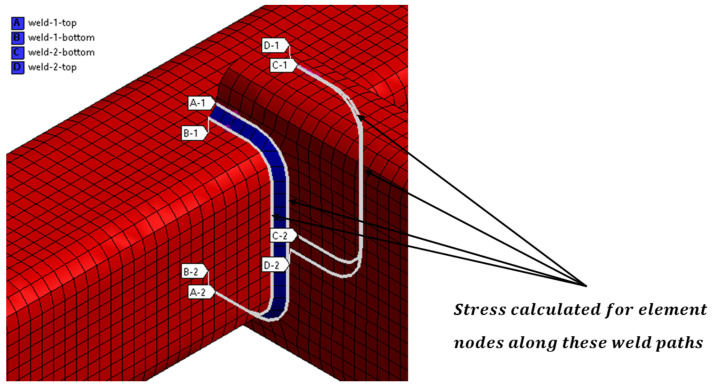
Evaluated modal EESS for the first ten modal responses of the structure along the weld path.

Figure 6 illustrates the plot of the modal EESS ${\emptyset_{i}}^{{∆S}_{s}}$ for the first ten modes for all twenty-six nodes along weld path 1A of the structure. The modal EESS values vary along the weld path, reflecting the different stress contributions from each mode.

**Figure 7:**
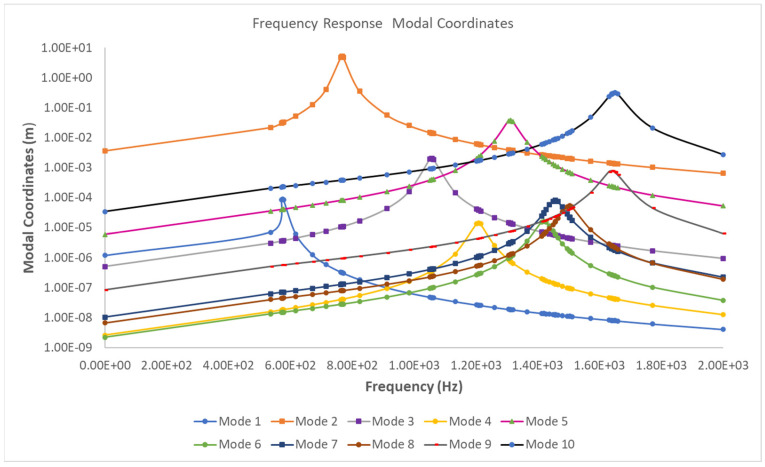
Evaluated frequency response coordinates for the first ten modal responses of the structure.

**Figure 8:**
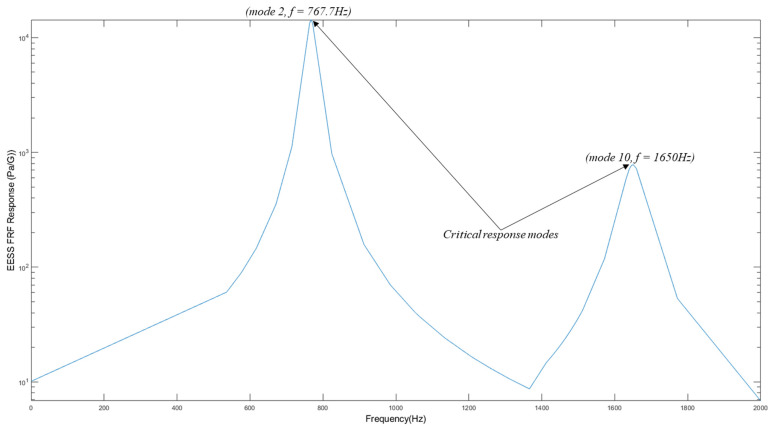
Maximum EESS FRF at node six on the weld toe paths.

**Figure 9:**
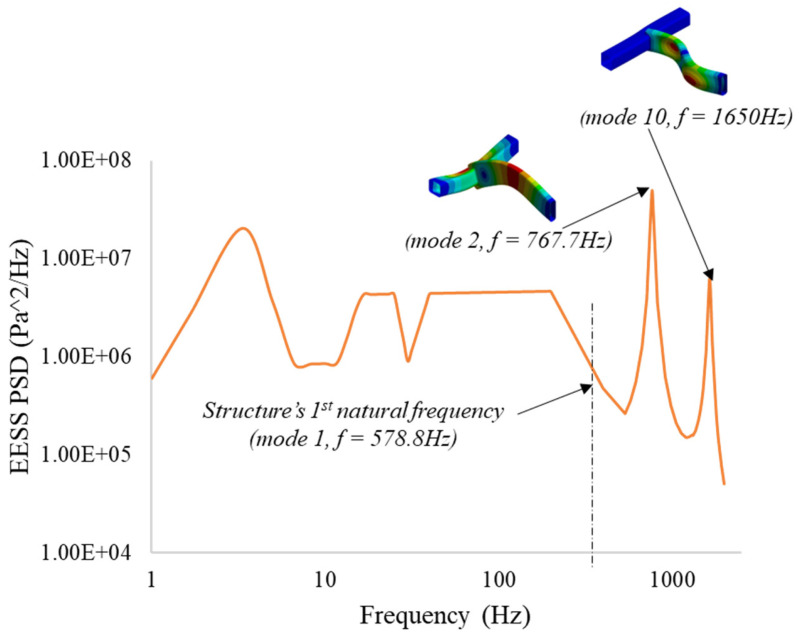
Maximum EESS response at node six: critical response at modes 2 and 10.

**Figure 10:**
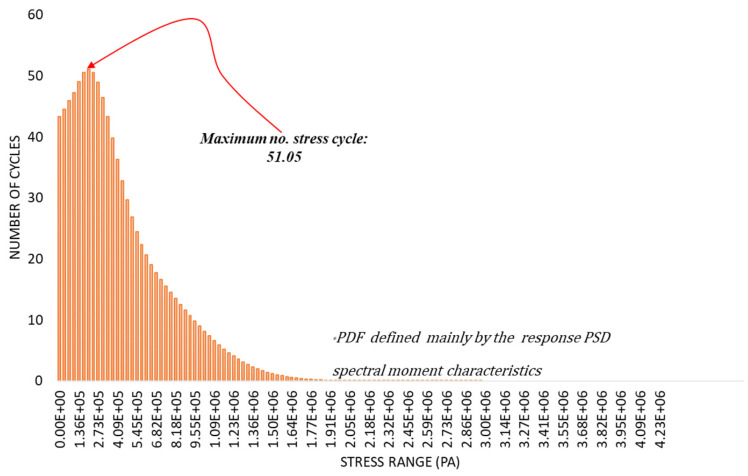
PDF of EESS amplitudes for the maximum EESS PSD.

Mesh element nodes six and twenty-five on the modelled weld joint path exhibit the highest modal EESS with stress values of 4645.97 Pa and 9279.13 Pa, respectively. These stress values correspond to modes 2 and 8, as seen in Figure 4, Figure 5, Figure 6, Figure 7, Figure 8, Figure 9 and Figure 10. These nodes play a significant role in the stress response and damage accumulation due to their higher mass fraction and proximity to critical locations. These modal EESS results, based on the structure’s modal responses, are considered in calculating the EESS FRF.

#### 3.2.2. Frequency Response of the Structure

The frequency response coordinates, as a function of frequency plot results at the weld joints, are presented in Figure 7. The frequency response coordinate plots showcase the structural response to the applied harmonic load across various frequencies for the first ten modal responses.

Figure 7 shows that the frequency response modal coordinates corresponding to modes 2 and 10 are the highest, stipulating the most sensitivity to the applied harmonic loads. Utilizing these FRF coordinates and the calculated modal stress results, the frequency response stresses are computed for all the nodes along the four weld paths of the structure. It is observed that modes with higher-frequency magnitudes than the applied load do not contribute significantly to the structural response, as the load magnitude remains constant. However, modes 2 and 10, representing in-plane displacement modes, significantly impact the stress and damage experienced by the structure. To identify the mesh node with the maximum EESS FRF response, further post-processing is conducted on the calculated EESS FRF results. Figure 8 illustrates the maximum stress frequency responses along weld path *1A* for mesh node six on the top weld path.

The maximum EESS response is observed at node six and is plotted separately below the stress frequency response plot. Notably, the peak stress frequency responses of 112,395 Pa/G and 32,672 Pa/G correspond to the natural frequencies of modes 2 and 10, respectively. These frequencies are extracted and incorporated into the maximum stress PSD and fatigue damage calculations. These findings emphasize the significance of considering the EESS FRF responses, including stress frequency responses and their association with the structural modes, in accurately predicting the fatigue damage and life of the structure.

#### 3.2.3. EESS PSD Response and PDF of the Structure

The maximum stress PSD responses $G^{{∆S}_{s}}\left(f\right)$ acting on node six along the top weld path of the structure, from which the damage and life evaluation is performed, are depicted in Figure 9. This stress response location corresponds to the same location as the maximum EESS frequency response. The maximum EESS PSD responses of $6.91\times 10^{8}$ ${Pa}^{2}/Hz$ and $1.47\times 10^{7}$ ${Pa}^{2}/Hz$ occur at two input excitation loading frequencies of 767.7 Hz and 1650 Hz, respectively, coinciding with the natural frequencies of the 2nd and 10th modes of the structure.

The spectrum results reveal that these two critical frequencies exhibit the most potent response to the load due to the maximum dynamic amplification effects around the structure’s second and tenth vibration modes, with natural frequencies of 767.7 Hz and 1650 Hz, respectively. These stresses, at the higher frequencies of the load excitation, contribute over 50% to the overall fatigue damage experienced by the structure. The structure’s response before the first natural frequency is primarily governed by its structural stiffness, resulting in a spectrum response following the load PSD. Multiple peak stresses within the natural frequencies are observed due to the applied loading. However, dynamic amplification effects do not influence stress responses before the first natural frequency of the structure. The peak stresses occur at node six, coinciding with the locations where the loading frequencies align with the structure’s natural frequencies.

The stress PDF of the structure, which defines the likelihood of occurrence for each calculated EESS amplitude, is presented in Figure 10.

The stress amplitude PDF, with a maximum number of stress cycles of 51.5 is shown in Figure 10. It is derived from the one-sided EESS PSD results across the excitation frequency range. From the figure, it can be observed that the ±1σ stress range values of $0–6.82\times 10^{5}$ Pa with a 68.3% likelihood of occurrence during the period of exposure to the load excitation, have the highest occurrence of repeated cycles, corresponding to a 32% chance of exceeding 50 cycles. These calculated stress range values, although considerably smaller compared to the maximum ±3σ stress range values of $6.83\times 10^{6}–2.86\times 10^{6}$ Pa, significantly contribute to the accumulated fatigue damage of the structure.

#### 3.2.4. Estimated Accumulated Fatigue Damage and Life of the Structure

**Figure 11:**
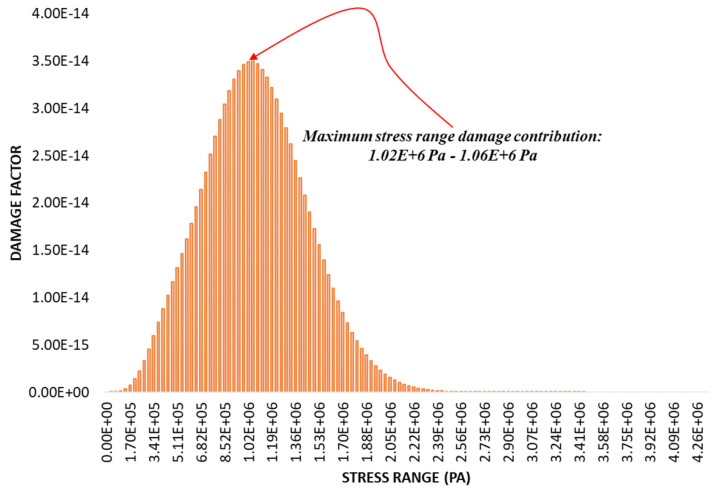
PDF of fatigue damage intensity.

The fatigue damage distribution, based on the contribution of each cyclic stress range, is plotted in Figure 11.

From the figure, it can be observed that 68.3% of the predicted accumulated fatigue damage induced in the structure is predominantly caused by the ±3σ stress range of $6.83\times 10^{5}–1.36\times 10^{6}\;Pa$. The ±3σ stress range is expected to occur only approximately 4.3% of the total exposure duration with a maximum number of cycles of eighteen, as shown in the stress distribution figure. The damage contribution of the ±1σ stress ranges is more than three times greater than that of the ±2σ stress ranges, which are predicted to occur for about 27.1% of the load exposure time.

**Figure 12:**
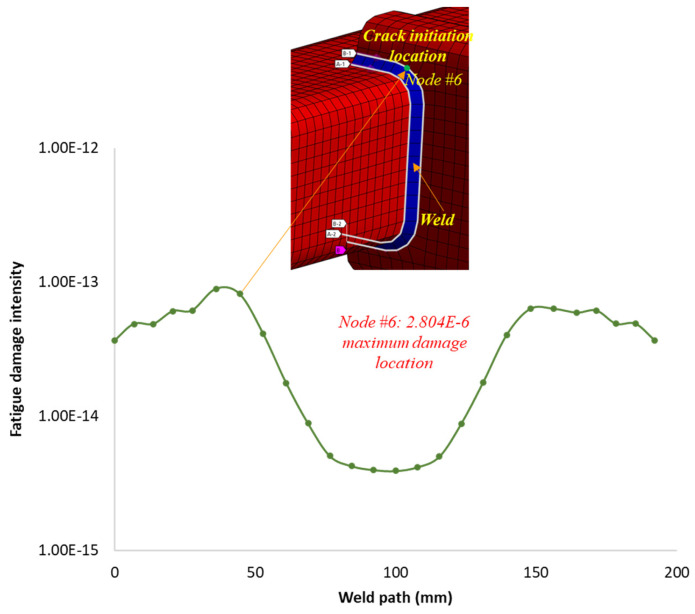
Predicted fatigue damage intensity along the weld toe path.

The variation in fatigue damage intensity along the weld paths is depicted in Figure 12. The region with the maximum cumulated estimated fatigue damage intensity, *D* = $2.804\times 10^{-6}$ per year, is observed at node six.

This region, as expected, corresponds to the most stressed area along the weld paths and is found to be critical, experiencing the highest level of damage. The minimum estimated fatigue life at node six, where the maximum damage occurs, is 99 years. This minimum calculated fatigue life is also considered as the estimated fatigue life of the structure under the considered load magnitude and exposure duration. Thus, it implies that the structure can sustain repeated random loading for a fatigue lifetime period of 99 years, based on a 90–95% confidence interval and a 98% probability of survival before the initiation of a damage crack.

## 4. Experimental Validation and Comparison with Existing Methods

The accumulated fatigue damage and life results of the welded structure obtained using the proposed frequency-domain fatigue analysis method are validated based on comparative analysis with fatigue test results reported in the literature [19] for the same structure and numerical analysis results obtained using the hotspot stress-based frequency-domain fatigue analysis method. The predicted fatigue damage crack initiation location on the structure’s weld joint using the proposed method is consistent with the area reported in the literature [19] under the same conditions, where uniaxial tensile stresses are applied perpendicular to the weld throat. These similarities in damage results validate the proposed method’s accuracy in predicting the fatigue damage crack initiation location and estimating its intensity for the considered random loading condition.

The fatigue life comparison uses the S–N curve parameters for the material and weld joint details described in Hobbacher [36]. The hotspot stress method analysis is performed for the same FEA model, PSD loading, and boundary conditions as the proposed method’s numerical application case.

The fatigue damage and life results obtained using the hotspot stress method are compared to the proposed EESS-based frequency-domain fatigue analysis method results in Table 1.

The predicted fatigue damage intensity factor of $2.804\times 10^{-6}$ and life of 99.09 years in Table 1 represent the values computed using the proposed EESS-based frequency-domain fatigue analysis method, as presented and discussed in Section 3.2.4. The values $2.2254\times 10^{-6}$ and 124.82 are the corresponding maximum fatigue damage intensity per year and the life of the structure, respectively, obtained using the hotspot stress method.

A 25.96% variation between the fatigue damage intensity and life calculated using the hotspot stress method and the proposed EESS-based frequency-domain fatigue analysis method can be observed in Table 1. The variation in the calculated results aligns with the outcomes of extensive verification analysis performed in this research to study the fatigue life discrepancies between the EESS and hotspot methods. The hotspot stress method’s conservative damage and life results can be attributed to the inclusion of the welded joints’ geometry and toe notch-induced stress concentration effects. These considerations are usually associated with using this method. However, in the proposed method, the weld joints’ highly mesh-sensitive stress concentrations have been characterized by the modal loading mode and modal EESS parameters, providing more accurate fatigue damage and life results.

## 5. Conclusions

This research has developed analytical equations and computational procedures that integrate the well-established Equilibrium Equivalent Structural Stress (EESS) fatigue evaluation formulations and structural dynamics analysis principles for a new EESS-based frequency-domain fatigue analysis approach. The proposed method evaluates the fatigue damage and life of welded structures that are subject to random loading.

The method, which predicts the damage and life of the structure before the crack initiation phase, is based on integrating the developed analytical equations for evaluating the modal EESS, EESS FRF, EESS PSD, and EESS amplitudes PDF, including the master S–N curve parameters and derivatives, C and h, into a frequency-domain fatigue evaluation framework. Using the mode superposition technique, these equations and computational procedures have been derived by considering and adapting the EESS formulations to account for the dynamic response characteristics of welded structures.

The viability and validity of the proposed method in accurately evaluating the stresses at weld joints and improving the accuracy of the predicted fatigue damage and life of welded structures have been demonstrated through numerical analysis and comparative analysis process using an RHS T-joint welded structure that is subjected to an irregular road surface profile-induced stationary random loading. Utilizing the master S–N fatigue curve parameters and derivatives and the calculated EESS PSD and amplitude results, the accumulated fatigue damage *D* due to the maximum EESS PSD was estimated to be $2.804\times 10^{-6}$ per year, and the corresponding predicted fatigue life of the structure before the crack initiation damage was 99 years. The predicted life is based on a 90–95% confidence interval and a 98% probability of survival before damage crack initiation.

The results of the proposed method are validated by comparison with the fatigue test data from the literature and outcomes obtained from the hotspot stress extrapolation-based methods. The predicted fatigue damage initiation locations on the structure align with the reported literature locations, affirming the accuracy of the proposed method. Additionally, the fatigue life prediction indicates an improvement of 26% compared to the hotspot method, thereby validating the effectiveness of the proposed approach. Notably, the proposed method achieves a maximum computational efficiency gain of 58% compared to the alternative hotspot stress-based fatigue analysis method.

This research contributes to the numerical fatigue design of welded structures subjected to random loading by introducing a significantly improved and effective frequency-domain fatigue assessment method. Identifying potential fatigue damage crack initiation locations and estimating the corresponding life with improved accuracy using the proposed method advances fatigue assessment in the design of welded structures by reducing the risk of premature failure, thereby mitigating economic and safety implications. As part of future works, the proposed equations and computational procedures will be further improved to account for the effects of the weld’s residual stresses on the structure’s estimated fatigue strength, damage, and lifetime. Also, the application of the proposed method will be extended to welded structures that are subjected to multiaxial and non-stationary random loadings.

## Figures and Tables

**Table 1:** EESS and hotspot frequency-domain methods predicted fatigue life comparison.

Frequency-Domain Fatigue Analysis Method	Damage Intensity Factor/Year	Fatigue Life (Years)	Variation (%)
EESS-based	$2.804\times 10^{-6}$	99.09	25.96
Hotspot stress-based	$2.2254\times 10^{-6}$	124.82	

## Data Availability

Data are contained within the article.
